# Design of S-Scheme CuInS_2_/CeO_2_ Heterojunction for Enhanced Photocatalytic Degradation of Pharmaceuticals in Wastewater

**DOI:** 10.1021/acs.langmuir.4c04175

**Published:** 2025-01-27

**Authors:** Olalekan C. Olatunde, Ibrahim Waziri, Damian C. Onwudiwe, Tunde L. Yusuf

**Affiliations:** † Department of Chemistry, School of Physical and Chemical Sciences, Faculty of Natural and Agricultural Sciences, North-West University, Mafikeng Campus, Private Bag X2046, Mmabatho 2735, South Africa; ‡ Department of Pure and Applied Chemistry University of Maiduguri, P.M.B., 1069, Maiduguri, Nigeria; § Department of Chemistry, Faculty of Natural and Agricultural Sciences, University of Pretoria, Private Bag X20, Hatfield, 0028, Pretoria, South Africa

## Abstract

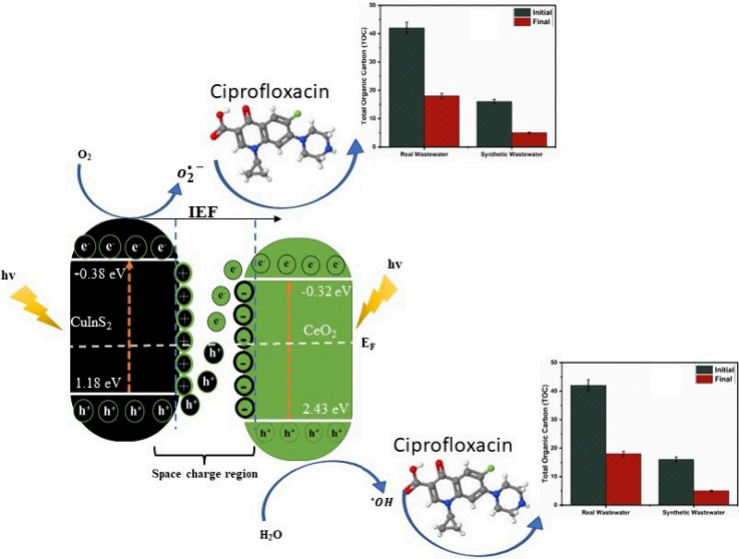

The release of common medications and illegal drugs into the environment could be potentially harmful to the ecosystem and hamper the behavior and growth of plants and animals. These pollutants gain access to water through sewage and factory discharges and have been found to exceed safety limits in water bodies. Therefore, there is an urgent need for improved wastewater purification systems. In this study, semiconductor-based heterojunction photocatalyst CuInS_2_/CeO_2_, synthesized through a facile solvothermal process, was explored for the photocatalytic degradation of ciprofloxacin, commonly used antibiotics. Studies on the electronic properties of the heterojunction revealed interfacial characteristics that were suitable for enhanced charge carrier separation and transport and a potential S-scheme charge transfer mechanism. The heterojunction achieved ∼90% efficiency for the degradation of CIP compared to 60% and 12% reported for CeO_2_ and CuInS_2_, respectively. This shows an improvement in the activity, which results from the improved charge carrier properties of the heterojunction. Further investigation of the charge transfer mechanism through radical scavenging experiments identified ^•^OH, O2^•–^, and h^+^ as active species contributing to the catalyst’s efficacy. Based on X-ray photoelectron spectroscopy analysis, a proposed S-scheme charge transfer mechanism was suggested for the CuInS_2_/CeO_2_ heterojunction. The findings indicate the potential of the CuInS_2_/CeO_2_ heterojunction as a promising photocatalyst for treating waste effluents from the pharmaceutical industry.

## Introduction

The presence of excessive amounts of pharmaceuticals in water pose a significant threat to both the sustainable development of the environment and human health.^1,2^ Recent reports have highlighted the persistence of pharmaceuticals in high concentrations within soil, plants, sediment, and water bodies.^3^ Pharmaceuticals enter the environment primarily through two main pathways: the discharge of pharmaceutical industry effluents into water streams and the improper disposal of large quantities of unused drugs.^4,5^ Ciprofloxacin (CIP), a widely used antibiotic known for its broad-spectrum antibacterial activity, is extensively utilized in human and veterinary medicine.^6,7^ With approximately 6 million prescriptions in the United States in 2018, CIP’s poor biodegradability, misuse, high stability, and hydrophilicity have contributed to its consistent detection and accumulation in water bodies.^8^ Studies have shown that CIP can induce various morphological alterations in the embryos of aquatic species and inhibit the growth of large freshwater producers.^9,10^ Given the reported toxicity levels associated with CIP, it is imperative to prioritize the removal of this antibiotic from pharmaceutical effluents and drinking water sources. This proactive approach is essential to safeguarding aquatic ecosystems and human health from the detrimental effects of ciprofloxacin contamination.

The presence of ciprofloxacin (CIP) and other antibiotics in water bodies indicates that current technologies, like adsorption and filtration, are not effectively tailored for eliminating this category of pollutants.^3^ As a result, there has been a shift toward investigating novel technologies as substitutes for traditional wastewater treatment systems. Heterogeneous photocatalysis, a type of advanced oxidation process, is recognized as a promising method for breaking down CIP into nontoxic inorganic compounds, all without the need for supplementary chemical oxidants.^11,12^ This facile and cost-effective technology involves the use of a semiconductor photocatalyst which, upon absorbing light energy greater than its bandap generates reactive oxygen species such as ^•^OH, O_2_^•–^, ^1^O_2_, and H_2_O_2_.^13^ However, the wide band gap of most typical photocatalysts, such as ZnO, CeO_2_, and TiO_2_, implies that the use of economically favorable solar energy is limited. Consequently, there is a pressing need to create photocatalysts with enhanced light absorption capabilities, either through the investigation of new materials or the modification of the electronic properties of current semiconductor photocatalysts.^14,15^

The formation of heterojunctions, which entails combining semiconductors with compatible band potentials, represents an optimal method for altering the electronic characteristics of photocatalysts.^16−18^ The discontinuity present at the interface between the two materials is responsible for the enhanced electronic properties of the heterostructure.^19^ The heterojunction benefits from improved charge carrier separation efficiency, which consequently enhances the charge carrier’s lifetime, leading to an overall improvement in photocatalytic performance.^20,21^ Generally, heterojunctions can be classified on three bases: (1) band configuration–which could be type-1 with a straddling gap, type-II with a staggered gap or type-III with a broken gap, (2) the types of semiconductor assembled, this could be p–p, n–n, or p–n, and (3) charge transfer mechanism, this could be type-II, p–n, S-scheme, or Z-scheme charge transfer mechanisms.^22,23^ In recent times, heterojunctions with the S-scheme charge transfer mechanism have gained much attention due to the maximizing of redox potential and prolonged charge carrier lifetime.^24^ The S-scheme heterojunction is formed by semiconductors with staggered band gaps. The component with the higher Fermi level is referred to as the reducing catalyst, while the oxidizing catalyst has a greater work function.^25^ Contact between the two semiconductors results in electron flow from the reducing catalyst to the oxidizing catalyst until Fermi level equilibrium is attained, leading to generation of the internal electric field (IEF) and consequent band bending. This band bending allows electrons from the oxidation photocatalyst to combine with holes in the reduction photocatalyst. This coloumbic interaction facilitates the separation of charge carriers while preserving electrons and holes with high redox potential.^26^ Therefore, efforts to selectively identify combinations of semiconductors with an appropriate band alignment for the S-scheme charge transfer mechanism have gained much attention recently.

While type-II and Z-scheme mechanisms have contributed significantly to improving photocatalytic performance, they exhibit several limitations. In type-II heterojunctions, the spatial separation of electrons and holes occurs across the interface, often leading to a reduced redox potential, which limits their application in reactions requiring high oxidation or reduction power. Additionally, the charge transfer process in type-II can result in increased recombination losses if the interface properties are not optimized. For Z-scheme mechanisms, the construction is more complex and often requires external electron mediators, which can increase costs and reduce stability.^27^ Furthermore, Z-scheme systems may suffer from efficiency losses due to the need for precise alignment of band potentials and potential side reactions introduced by the mediators. These challenges have driven the pursuit of alternative mechanisms, such as S-scheme heterojunctions, which aim to overcome these shortcomings by simultaneously enhancing charge separation and preserving redox potential.^28^

Various semiconductors, including metal oxides, are currently being explored as catalysts for wastewater treatment because of their environmental friendliness and ease of synthesis.^29^ Cerium oxide (CeO_2_) has been extensively explored as a photocatalyst in wastewater treatment due to its high chemical resistance. However, its wide band gap energy range of 2.6–3.2 eV makes it unsuitable for visible light absorption.^30^ Compositing CeO_2_ with a narrow band gap semiconductor with visible-light responsiveness like CuInS_2_ is a facile and effective way to enhance the photocatalytic activity of CeO_2_.^31^ CuInS_2_ a typical I–III–VI_2_ semiconductor, is known for its narrow band gap of 1.52 eV and its absorption in the whole visible range of the solar spectrum, allowing it to exploit the solar spectrum maximally.^32^ Therefore, a heterojunction comprising CeO_2_ and CuInS_2_ could possess suitable optical, electronic, and charge transfer characteristics that could enhance its activity for antibiotics degradation.

Consequently, this study reports the facile synthesis of a novel CuInS_2_/CeO_2_ heterojunction and its photocatalytic activity for antibiotic degradation. The formation of the novel heterojunction was confirmed through various characterization techniques, and the electronic property characterization suggested the potential of the heterojunction for S-scheme charge transfer. The mechanism of charge transfer was further elucidated through the information obtained from XPS analysis and radical scavenging experiments. This work has, therefore, shown how a selective combination of semiconductor materials in the formation of heterojunctions could be an important route in obtaining highly efficient photocatalysts for dealing with the presence of pharmaceuticals in water. Also, a clear understanding of the mechanism presented offers a clear path for the optimization of the catalytic process.

## Experiment

### Chemicals

All chemicals and reagents were purchased from commercial sources.

#### Synthesis of Nanomaterials

##### Synthesis of CeO_2_

CeO_2_ nanorods were synthesized by using a hydrothermal method. In this approach, 1 g of cerium nitrate hexahydrate (Ce(NO_3_)_3_·3H_2_O) was dissolved in 50 mL of deionized water and added to a stirred solution of 9.2 g of sodium hydroxide in 100 mL of deionized water. The resulting mixture was then transferred to an autoclave and maintained at 100 °C for 24 h. After the reaction, the product was thoroughly washed with deionized water and dried at 70 °C for 24 h, yielding CeO_2_ nanorods.

### Synthesis of CuInS_2_/CeO_2_

The CuInS_2_/CeO_2_ heterostructures were synthesized by using a solvothermal method. Initially, 0.2 g of CeO_2_ was dispersed in DMF and stirred for 15 min. Subsequently, stoichiometric amounts of copper chloride(1 mmol), indium chloride(1 mmol), and thioacetamide (2 mmol) were added to the solution, which was then stirred for an additional 30 min. The resulting mixture was transferred to an autoclave and heated at 180 °C for 12 h. After the reaction, the mixture was thoroughly washed with deionized water and dried overnight at 70 °C. The heterojunctions were labeled as 0.5CuInS_2_/CeO_2_, 1.0CuInS_2_/CeO_2_, 1.5CuInS_2_/CeO_2_, and 2.0CuInS_2_/CeO_2_, corresponding to mole ratios of 5%, 10%, 15%, and 20%, respectively. To synthesize pristine CuInS_2_, the same procedure was followed, but without the addition of CeO_2_.

#### Evaluation of Antibiotics Degradation

A 100 W xenon solar lamp was utilized as the light source for the oxidation of antibiotics. The lamp was positioned 10 cm above the solution surface. The photooxidation of ciprofloxacin (5 mg/L, 50 mL), sulfamethoxazole (5 mg/L, 50 m), and tetracycline (5 mg/L, 50 mL) was examined under these conditions. A catalyst (30 mg) was dispersed in the solution containing the pollutants and stirred in darkness for 30 min to achieve adsorption–desorption equilibrium. After this period, the light was switched on, and 3 mL samples were taken from the solution at specific intervals. These samples were centrifuged at 7000 rpm for 10 min to effectively separate the catalyst. The supernatant was then analyzed by using a UV–visible spectrophotometer.

The photocatalytic degradation efficiency was calculated using the following equation:
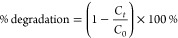
1where *C*_0_ (mg/L) represents the initial concentration of antibiotics, while *C*_*t*_ (mg/L) represents the concentration of antibiotics at a particular time (*t*).

The recyclability of the catalyst was evaluated by washing it three times with deionized water and drying it at 80 °C for 5 h before reusing it in subsequent experiments. All photo-oxidation experiments were conducted in triplicate. Also, the experiment was conducted using real wastewater from a wastewater treatment plant (Daspoort WWTP, Pretoria, South Africa).

#### Determination of Reactive Species

To identify the primary reactive species involved, scavenger experiments were conducted using different quenchers, including acrylamide, isopropanol (IPA), and sodium EDTA, at concentrations of 0.2 mmol/L, which targeted superoxide radicals, hydroxyl radicals, and holes, respectively

## Results and Morphology

### Structure and Morphology

**Figure 1:**
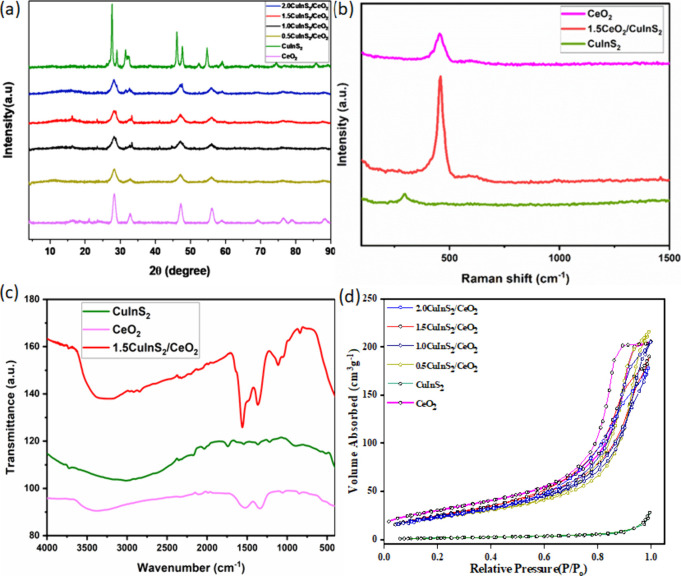
(a) XRD spectra of CeO_2_, CuInS_2_, and CuInS_2_/CeO_2_, (b) Raman spectra of CeO_2_, CuInS_2_, and CuInS_2_/CeO_2_, and (c) FTIR spectra of CeO_2_, CuInS_2_ and 1.5CuInS_2_/CeO_2_, and (d) BET plot for CeO_2_, CuInS_2_, and CuInS_2_/CeO_2_ heterojunctions at different weight ratios.

Figure 1a shows the XRD spectra, Raman spectra, FTIR spectra, and BET plots of CeO_2_, CuInS_2_ and CuInS_2_/CeO_2_ heterostructures. The diffraction pattern of CeO_2_ matches the face-centered cubic phase of CeO_2_, as referenced in JCPDS No: 04–006–2393.^33^ For CuInS_2_, the XRD spectra were consistent with the tetragonal chalcopyrite phase of CuInS_2_ with lattice parameters *a* = *b* = *c* = 5.51 Å, *c* = 11.32 Å, and α = β = γ = 90° (JCPDS No. 47–1327).^34^ The XRD pattern of CuInS_2_/CeO_2_ showed peaks similar to CeO_2_ only, showing that the crystal structure of CeO_2_ was sustained after doping. The low doping content of CuInS_2_ accounted for the absence of its peak in the XRD pattern of CuInS_2_/CeO_2_ heterojunctions.^35^

Figure 1b shows the Raman spectra for CeO_2_, CuInS_2_, and CuInS_2_/CeO_2_. The spectra for CeO_2_ only showed one allowed *T*_2_*g* mode due to its typical cubic crystal structure.^36^ The single peak observed at 465 cm^–1^ is consistent with reports in the literature and is considered the first-order scattering arising from the Ce–O–Ce symmetric vibration.^37^ The Raman spectra of CuInS_2_ showed a characteristic peak of the A1 vibrational mode at around 295 cm^–1^.^38^ The *E*_3_ longitudinal and *E*_1_ transverse modes at around 240 and 340 cm^–1^ could also be observed.^39^ The absence of any additional peak in the Raman spectra confirmed the phase purity of the synthesized CeO_2_ and CuInS_2_ nanoparticles. No Raman lines due to CuInS_2_ were observed in the spectra of the CuInS_2_/CeO_2_ heterojunction, which is consistent with the observations from the XRD spectra. However, the CeO_2_ peak was observed to be more intense compared to the pristine CeO_2_. Raman band peak intensity has been reported to be influenced by several factors, such as strain, grain size and morphology.^40^ Therefore, the heterojunction formation between CeO_2_ and CuInS_2_ could have possibly influenced the microstructural properties of the obtained CuInS_2_/CeO_2_ heterojunction.

In the FTIR spectra of CuInS_2_ (Figure 1c), vibrations arising from S–In and Cu–In bonds were observed at about 1720 and 1450 cm^–1^, respectively.^41^ The FTIR spectra of CeO_2_ showed the typical Ce–O stretching vibration at around 740 cm^–1^. The O–H stretching vibration was observed at 1625.20 and 1450.5 cm^–1^. The broad O–H peak at ∼3500 cm^–1^ could be assigned to O–H stretching and bending vibrations of water on CeO_2_ surface.^42^ The CuInS_2_/CeO_2_ heterojunction showed peaks similar to those of CeO_2_, but with an increased intensity. This could also confirm the alteration of the microstructural property as observed in the Raman spectra.

To further explore the microstructural properties of the materials, BET analysis of the material was carried out, as shown in Figure 1d. The materials had similar N_2_ adsorption–desorption isotherms, showing a sharp increase in adsorption at a *P*/*P*_o_ value close to 1.0. This is consistent with type IV isotherm and reveals the presence of mesopores^43^ The surface area, pore volume and pore size distribution estimated for the materials are shown in Table 1.

**Table 1:** BET Surface Area, Pore Volume, and Pore Size of Photocatalyst Material

	CeO_2_	CuInS_2_	0.5CuInS_2_/CeO_2_	1.0CuInS_2_/CeO_2_	1.5CuInS_2_/CeO_2_	2.0CuInS_2_/CeO_2_
BET surface area (m^2^/g)	112.3	6.67	84.6	91.7	96.3	90.0
pore volume (cm^3^/g)	0.317	0.043	0.334	0.315	0.294	0.275
pore size (nm)	11.30	25.89	15.76	13.73	12.22	12.53

**Figure 2:**
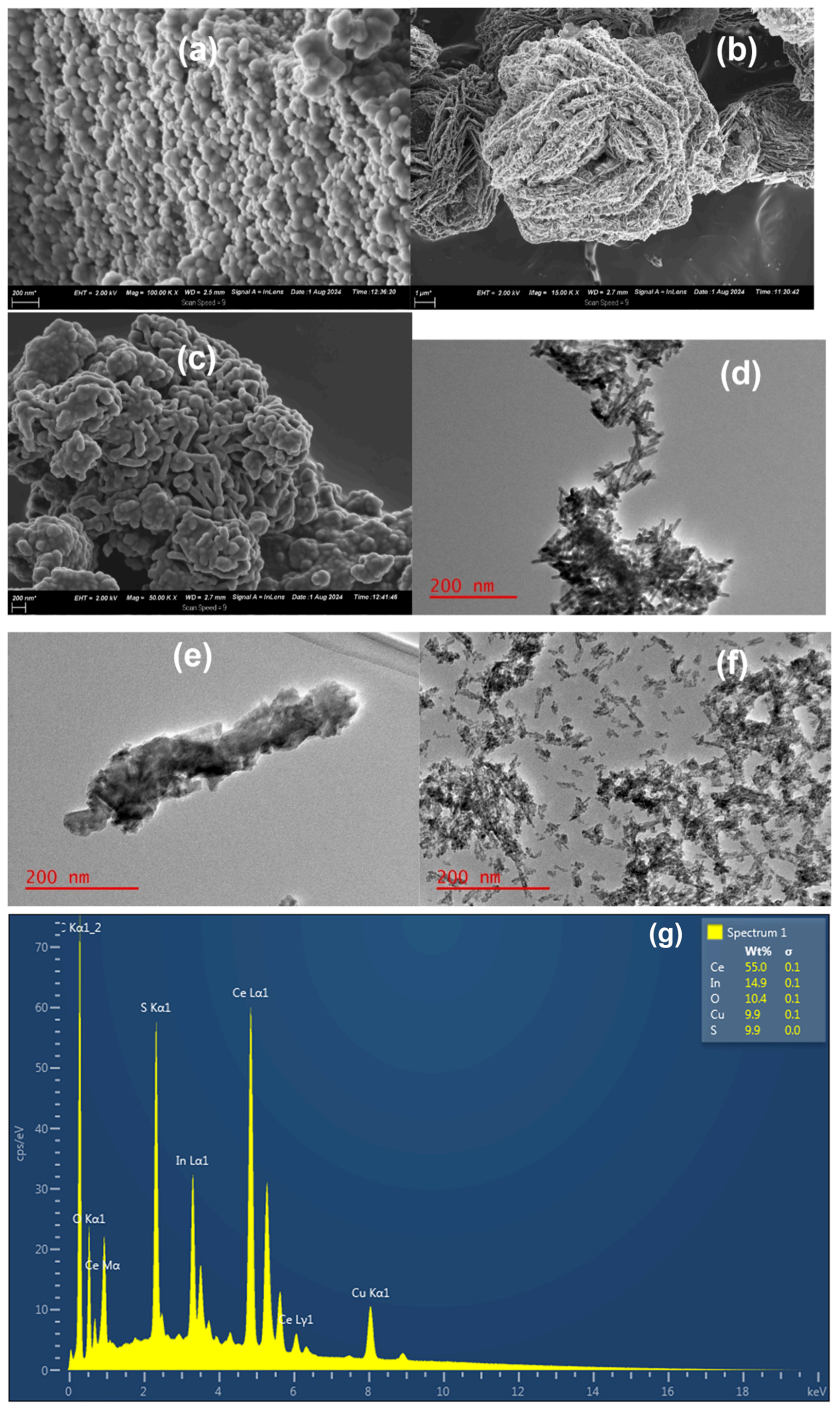
SEM micrographs of (a) CeO_2_, (b) CuInS_2_, and (c) 1.5CuInS_2_/CeO_2_. The TEM micrographs of (d) CeO_2_, (e) CuInS_2_, (f) 1.5CuInS_2_/CeO_2_, and (g) EDS of 1.5CuInS_2_/CeO_2_.

The SEM and TEM images of CeO_2_, CuInS_2_, and CuInS_2_/CeO_2_ are depicted in Figure 2a–f. The SEM image of CeO_2_ (Figure 2a) reveals a highly agglomerated, uniform, and granular morphology. In contrast, CuInS_2_ (Figure 2b) exhibits a more aggregated morphology with larger, clustered granules, displaying a rougher surface and more pronounced intergranular spaces compared to CeO_2_. In the CuInS_2_/CeO_2_ heterojunction (Figure 2c), CeO_2_ seems to predominate in the morphology, resulting in a less uniform structure, likely due to the integration of CuInS_2_ into the matrix of CeO_2_. This induced structural modification has the potential to influence the catalytic activity of the formed heterojunction when compared to the pristine materials. The TEM image of CeO_2_ (Figure 2d) shows irregular, quasi-spherical shaped particles, whereas, elongated rod-like shaped CuInS_2_ nanoparticles were observed (Figure 2e). The TEM image of CuInS_2_/CeO_2_ (Figure 2f) displayed a more dispersed, and interconnected network of CeO_2_ and CuInS_2_. The interaction between the two materials could lead to enhanced properties for improved photocatalytic activity. The EDS spectra of CuInS_2_/CeO_2_ (Figure 2g) showed the successful formation of the heterojunction with the presence of peaks due to Cu, In, S, Ce, and O.

**Figure 3:**
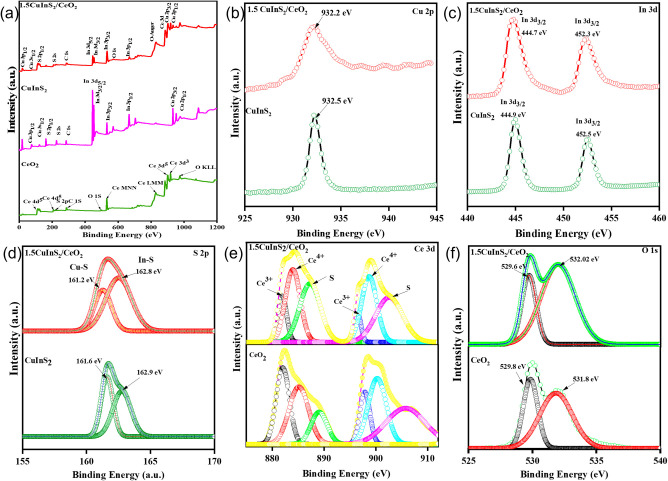
(a) Survey scan of CeO_2_, CuInS_2_, and 1.5CuInS_2_/CeO_2_; (b) High resolution scan of Cu 2p, (c) In 3d, (d) S 2p, (e) Ce 3d, (f) and O 1s.

The compositions of the CeO_2_, CuInS_2_, and CuInS_2_/CeO_2_ were further characterized by XPS, as shown in Figure 3. The survey spectra (Figure 3a) confirmed the presence of the constituent elements of CuInS_2_ and CeO_2_, while the spectra of CuInS_2_/CeO_2_ confirmed the coexistence of Cu, In, S, Ce, and O elements, which confirms the heterojunction formation. The Cu 2p, In 3d, and S 2p core levels were employed in determining the valence states of the elements in CuInS_2_. Figure 3b shows the high-resolution XPS spectra of Cu 2p. The peak observed at 932.5 eV belongs to the Cu 2p_3/2_.^44^ From the survey scan, the split between the Cu 2p_1/2_ and Cu 2p_3/2_ peak is about 22 eV. This doublet is assigned to the Cu^+^ in CuInS_2_.^45^ The high-resolution spectra of In 3d are shown in Figure 3c. The XPS peaks of In 3d_3/2_ and In 3d_5/2_ were observed at 452.3 and 444.9 eV. With a peak separation of 7.4 eV, this confirms that In exists as In^3+^.^46^Figure 3d shows the S 2p peaks observed at 161.6 4 and 162.9 eV for Cu–S and In–S, respectively. The splitting of the peak into the S 2p_1/2_ and sp_3/2_ doublets is due to spin–orbit coupling.^47^ The energy difference between the doublet was found to be 1.1, consistent with S^2+^.^48^ The Ce 3d spectra of CeO_2_ show characteristic peaks at 888.1 and 907.5 eV from Ce^3+^, while the peaks at 883.7, 901.3, and 917.5 eV correspond to the Ce^4+^ oxidation state.^49^ The O-1s spectrum showed peaks at 529.6 and 532.0 eV, which could be deconvoluted to CeO_2_ lattice oxygen and surface-adsorbed oxygen, respectively.^50^ The spectra of CuInS_2_/CeO_2_ gave evidence of charge carrier migration across the interface between CeO_2_ and CuInS_2_, as shifts in the binding energy of the elements could be observed.

#### Optical Properties

**Figure 4:**
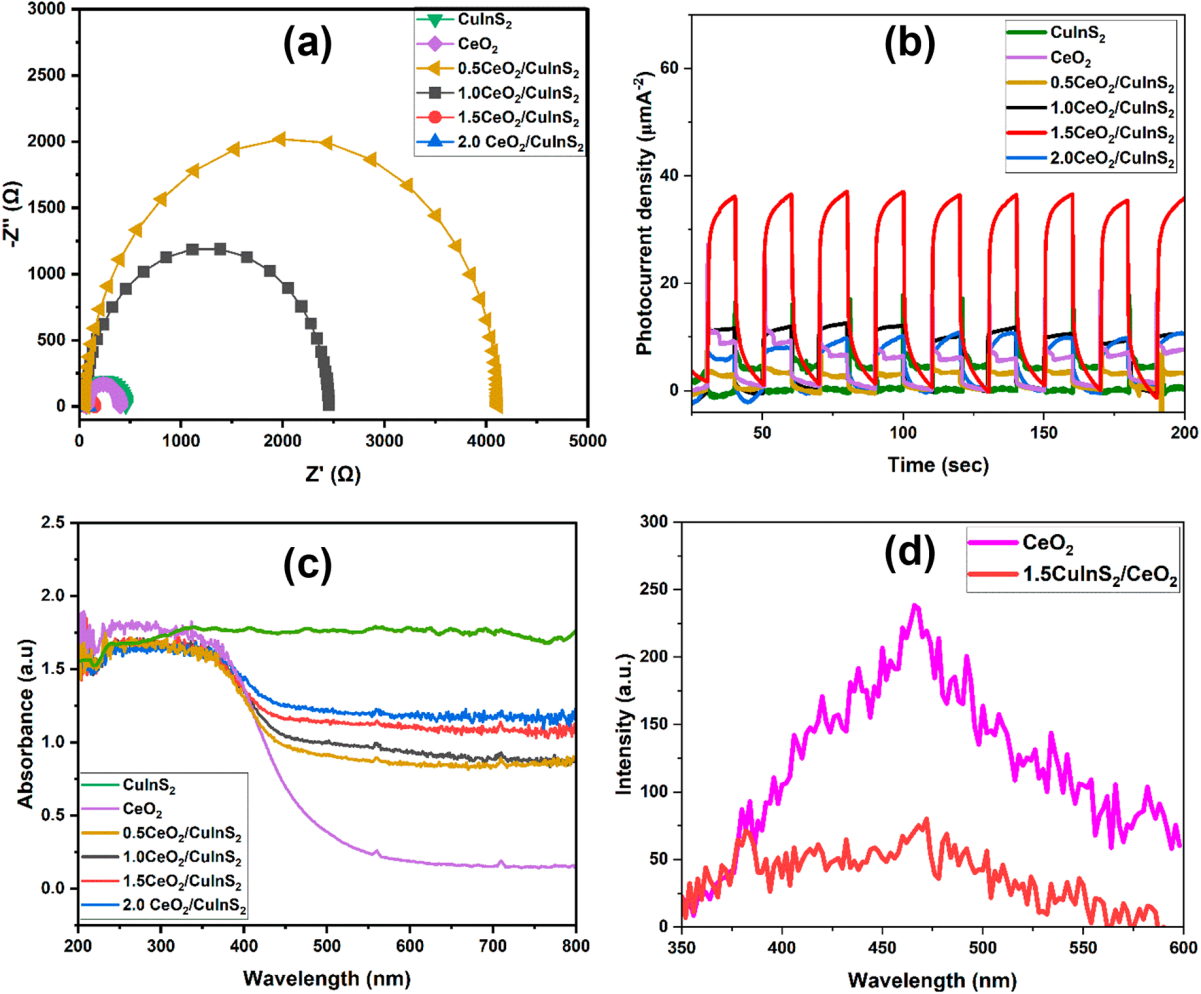
(a) EIS spectra of CeO_2_, CuInS_2_, and CuInS_2_/CeO_2_ heterojunctions; (b) Photocurrent density of CeO_2_, CuInS_2_, and CuInS_2_/CeO_2_ heterojunctions; (c) UV–vis spectra of CeO_2_, CuInS_2_, and CuInS_2_/CeO_2_ heterojunctions; (d) Photoluminescence spectra of CeO_2_, CuInS_2_, and 1.5CuInS_2_/CeO_2_ (excited at 325 nm).

Charge carrier characteristics such as lifetime, recombination rate, and diffusion length are significant parameters in the design of photocatalytic nanomaterials.^51^ Therefore, the modulation of charge carrier dynamics is significant in enhancing the photocatalytic activity of catalytic materials. To explore the charge carrier properties of CeO_2_, CuInS_2_, and CuInS_2_/CeO_2_ heterojunctions, the electrochemical impedance spectroscopy (EIS) and current density measurements were done. The EIS shows a visible semicircle, which gives important information about the charge carrier transport, separation and resistance of the light-induced charge carriers. The EIS spectra for CeO_2_, CuInS_2_, and CuInS_2_/CeO_2_ heterostructures are shown in Figure 4a. The charge-transfer resistance at the electrode–electrolyte interface is inversely correlated to the radius of the EIS arc.^52^ The EIS plot showed the CuInS_2_/CeO_2_ heterojunction had a lower charge transfer resistance compared to CeO_2_ and CuInS_2_, signifying the improved separation of photogenerated charge carriers by heterojunction formation. This improved charge carrier separation is significant for enhanced photocatalytic activity as the photogenerated carriers become available for inducing pollutant degradation.^53^ Photocurrent charge density generally benefits from enhanced charge carrier separation and transport. The photocurrent charge densities of CeO_2_, CuInS_2_ and CuInS_2_/CeO_2_ heterostructures were explored by plotting current density (*I*) against potential (*V*) under both dark and illumination. Figure 4b, shows that the 1.5CuInS_2_/CeO_2_ heterojunction showed significantly improved photocurrent density compared to the pristine materials. The photocurrent density of 1.5CuInS_2_/CeO_2_ was almost double that of CeO_2_ and CuInS_2_, showing the importance of heterojunction formation and the composition of the material. The spike in the photocurrent response observed during the illumination phase, infers the charge separation between the sample and electrolyte. The immediate response implies an efficient separation of the photogenerated electron–hole pair, which implies improved photocatalytic activity.^54^ In the dark, hole migration in the electrolyte and electron transfer from the semiconductor via the FTO, result in fast photocurrent loss. Charge carrier recombination could also, account for the photocurrent loss during the dark phase.^55^ Remarkably, the 1.5CuInS_2_/CeO_2_ showed a relatively constant photocurrent density with time, which shows its stable charge carrier generation and transport.^56^ The UV–vis absorption spectroscopy results for CeO_2_, CuInS_2_, and CuInS_2_/CeO_2_ heterojunctions are shown in Figure 4c. It was observed that the light absorption intensity of the heterojunctions was intermediate between those of CeO_2_ and CuInS_2_. A slight blue shift in the absorption edge of CuInS_2_/CeO_2_ heterojunctions was observed compared to CuInS_2_, suggesting the alteration of the electronic structure of CuInS_2_ due to CeO_2_ incorporation. Furthermore, as shown in Figure 4d, the 1.5CuInS_2_/CeO_2_ heterojunction showed a lower photoluminescence peak intensity compared to CeO_2_ and CuInS_2_, confirming the improved charge carrier separation in the heterojunction compared with the pristine materials.

Mott–Schottky curves of pristine CeO_2_, CuInS_2_, and CuInS_2_/CeO_2_ heterojunctions were measured to determine the flat-band potentials and the nature of the semiconductivity of the materials. The flat band potentials (*V*_fb_) for the materials were estimated according to the Mott–Schottky equation:
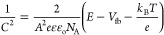
where *C* is the interfacial capacitance, *A* is the electrode surface, *e* is the electronic charge, ε is the dielectric constant, ε_o_ is the free space permittivity, *N*_A_ is the carrier concentration, *E* is the applied voltage, *k*_B_ is the Boltzmann constant, and *T* is the absolute temperature.

**Figure 5:**
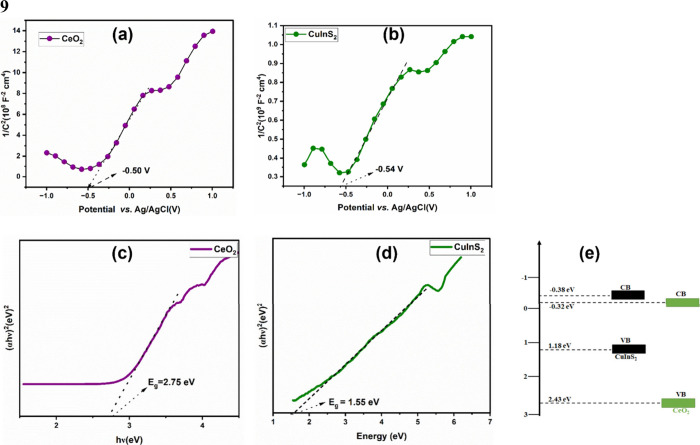
Mott–Schottsky plot for (a) CeO_2_ and (b) CuInS_2_ and Tauc plot for (c) CeO_2_, (d) CuInS_2_, and (e) band alignment in the 1.5CuInS_2_/CeO_2_ heterojunction.

The *V*_fb_ is estimated from the Mott–Schottsky by extending the linear portion of the curve to the *X*-axis (Figure 5a,b). The *V*_fb_ values for CeO_2_ and CuInS_2_ were −0.50 and −0.56 V, respectively. Furthermore, the positive slope of both curves shows the n-type conductivity of the pristine materials.^57^ The VB and CB positions of CuInS_2_ and CeO_2_ were estimated using the equation:
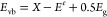

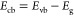
where *X* is the absolute electronegativity, *E*^*e*^ is the free electrons energy on the hydrogen scale (4.5 eV), and *E*_g_ is the materials band gap energy. The value of *X* for CuInS_2_ and CeO_2_ is 4.9^58^ and 5.56.^59^Figure 5c,d shows the Tauc plots from which the *E*_g_ of CeO_2_ and CuInS_2_ was determined. The *E*_g_ for CeO_2_ and CuInS_2_ was evaluated to be 2.75 and 1.56 eV, respectively, which are in agreement with reports in literature.^60,61^ The *E*_vb_ for CuInS_2_ and CeO_2_ was 1.18 and 2.44 eV, while the *E*_cb_ was estimated to be −0.38 and −0.32 eV, respectively. Based on the estimated band edge potentials, the band alignment for the CuInS_2_/CeO_2_ heterojunction is proposed as shown in Figure 5e.

### Degradation of Antibiotics

**Figure 6:**
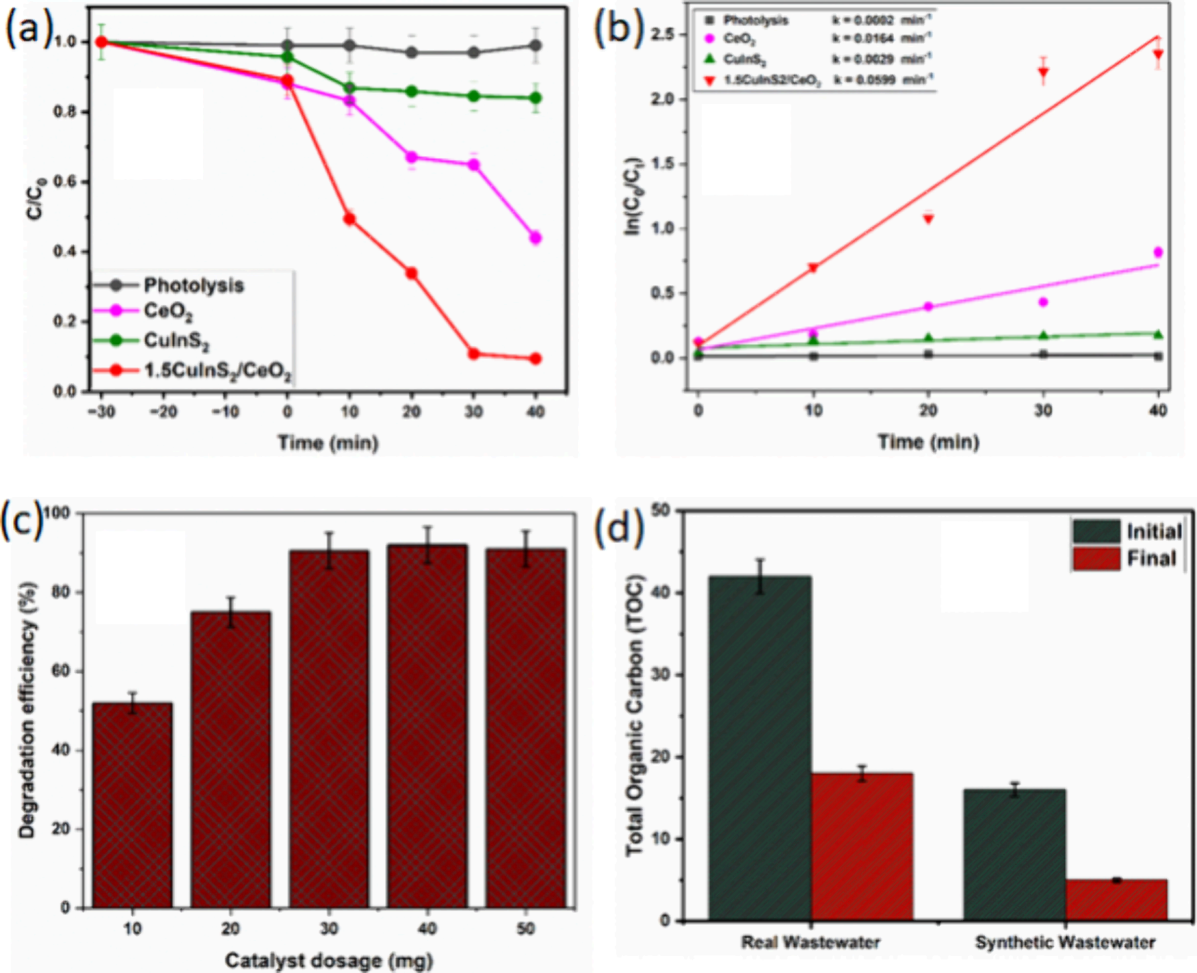
(a) Photocatalytic degradation profile of CIP by CeO_2_, CuInS_2_, and CuInS_2_/CeO_2_; (b) Pseudo-first order kinetics plot for CIP degradation; (c) Effect of catalyst dosage on the degradation of CIP; (d) TOC removal by CuInS_2_/CeO_2_ in synthetic and real water samples.

The photocatalytic activities of CeO_2_, CuInS_2_, and 1.5CuInS_2_/CeO_2_ heterojunction were investigated for the reduction of CIP (Figure 6a). To achieve the adsorption and desorption equilibrium, the reaction was stirred in the dark for 30 min before illumination. From the dark phase reaction, CeO_2_ and CuInS_2_/CeO_2_ were observed to show similar adsorption level (∼10%) compared to CuInS_2_. During the light phase of the experiment, the CIP was observed to undergo rapid reduction in the presence of CuInS_2_/CeO_2_, with ∼90% degradation achieved in 40 min. This showed a rapid enhancement in photocatalytic activity compared to that of pristine CeO_2_ and CuInS_2_, which achieved 60% and 12% efficiency, respectively. This showed that the heterojunction capacity was enhanced by factors of 1.5 and 7.5 in comparison to CeO_2_ and CuInS_2_. This enhanced photocatalytic activity could be attributed to the improved charge carrier separation achieved by the formation of a heterojunction at the CeO_2_ and CuInS_2_ interface. Figure 6b shows the pseudo-first kinetic fit for the degradation process. The 15CuInS_2_/CeO_2_ heterojunction showed a higher reaction rate constant (*k*), which is about 20 and 3.6 times higher than those of CuInS_2_ and CeO_2_, respectively. Table 2 shows that CuInS_2_/CeO_2_ heterojunction compared favorably with reported CeO_2_-based heterojunctions in literature.

**Table 2:** Comparison of CIP Degradation by a Previously Reported CeO_2_-Based Heterojunction Photocatalyst

catalysts	catalyst dosage	Cr(VI) parameters	degradation efficiency (%)	rate	ref
CeO_2_/ZnO	25 mg	15 mg/L	60	0.0130 min^–1^	(62)
		100 mL			
		60 min			
Cd0.5Zn0.5S/CeO_2_	30 mg	5 mg/L	86	0.0454 min^–1^	(63)
		50 mL			
		30 min			
CeO_2_–Ag/AgBr	50 mg	10 mg/L	93	0.0201 min^–1^	(64)
		50 mL			
		120 min			
MoS_2_/CeO_2_	50 mg	10 mg/mL	89	0.017 min^–1^	(65)
		100 mL			
		120 min			
1.5CuInS_2_/CeO_2_	30 mg	5 mg/mL	90	0.056	this study
		50 mL			
		45 min			

To further study the practical applicability of the CuInS_2_/CeO_2_ heterojunction, the effect of catalyst dosage and its effectiveness in real water samples were studied. The catalyst showed a concentration-dependent activity, with optimal activity achieved at a 40 mg catalyst dosage (Figure 6c). Charge carrier production correlates directly with the catalyst dosage; however, excess catalyst could negatively impact the photocatalytic process by hindering the penetration of light into the pollutant solution.^66^ The total organic carbon (TOC) removal by the heterojunction in real water samples and synthetic wastewater was evaluated to investigate how the presence of organic matter influences the photocatalytic activity of the material. Figure 6(d) shows the TOC removal in real water samples and synthetic wastewater. The TOC removal in the real water sample reached 39.0%, while in the synthetic wastewater, the TOC removal was 33.3%. This shows the efficiency of the CuInS_2_/CeO_2_ heterojunction was not inhibited by the presence of organic matter in the wastewater and could be widely applied to a wide range of wastewater sources.

**Figure 7:**
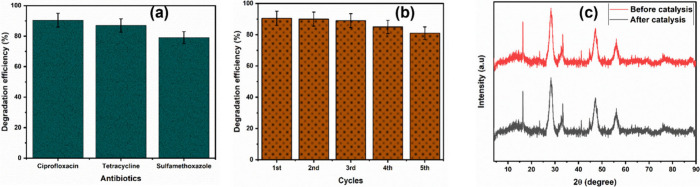
(a) Degradation efficiency of 1.5CuInS_2_/CeO_2_ for other antibiotics; (b) Stability test for 1.5CuInS_2_/CeO_2_; (c) XRD spectra of 1.5CuInS_2_/CeO_2_ before and after 5 catalytic cycles.

Furthermore, the efficiency of the heterojunction for the photocatalytic oxidation of two other pharmaceuticals was explored (Figure 7a). The catalyst showed high efficiencies of 84% and 78% for tetracycline and sulfamethoxazole, respectively. This confirms the potential wide-range application of the CuInS_2_/CeO_2_ heterojunction. The catalyst recyclability test was carried out to study the stability of the CuInS_2_/CeO_2_ heterojunction. Figure 7b shows the catalyst still achieved about 79% CIP degradation after five (5) application cycles. Figure 7c shows the XRD pattern of the CuInS_2_/CeO_2_ catalyst before and after the stability test, with no observable change in the structure of the catalyst, confirming its stability.

#### Degradation Mechanism

**Figure 8:**
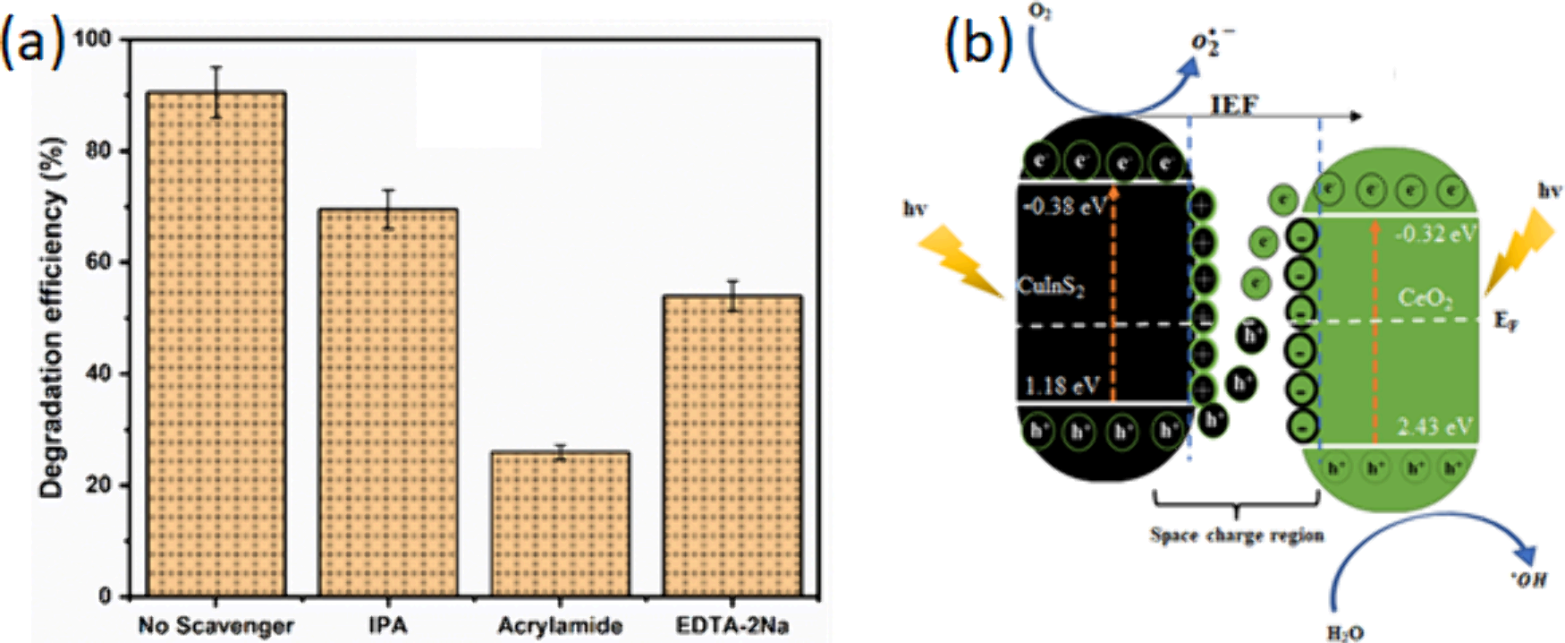
(a) Radical scavenging experiment; (b) Proposed S-scheme charge transfer mechanism for CuInS_2_/CeO_2_ heterojunction.

To investigate the mechanism of action of the CuInS_2_/CeO_2_ heterojunction, radical scavenging studies were carried out. The degradation was carried out in the presence of isopropyl alcohol (IPA), acrylamide, and EDTA-2Na as hydroxyl (^•^OH), superoxide (O_2_^•–^), and hole (h^+^) scavenger. The radical scavenging experiment (Figure 8a) showed that the three radical species were involved in the degradation of CIP. However, the radical O_2_^•–^ was observed to have the most significant influence on the degradation activity of CuInS_2_/CeO_2_. The radical scavenging experiment thus confirms the effective separation of photogenerated charge carriers and their availability for inducing the degradation of CIP.

Theoretically, the conduction bands of CeO_2_ and the VB of CuInS_2_ are not capable of independently generating O_2_^•–^ and ^•^OH. This is due to the standard reduction potential of O_2_/O_2_^•–^ (−0.33 eV) and ^•^OH/H_2_O (+2.31), being higher than the CB and VB of CeO_2_ and CuInS_2_, respectively. Therefore, the generation of O_2_^•–^ and ^•^OH during the degradation process, confirms the preservation of the photogenerated e^–^ and h^+^ on the CB of CuInS_2_ and VB of CeO_2_, respectively. The proposed band alignment in the CuInS_2_/CeO_2_ heterojunction showed staggering of the band edges of the materials. This radical species generation is consistent with the S-scheme charge transfer mechanism.

Generally, in S-scheme charge transfer mechanisms, a decrease or increase in electron density before or after contact is often observed in the material.^67^ This is often evaluated from the XPS spectroscopy spectrum. The Ce 3d high-resolution spectrum of pristine CeO_2_ showed redshift in CuInS_2_/CeO_2_ heterojunction from 883.7 to 882.4 eV, 888.9 to 888.3 eV, and 901.3 to 889.3 eV. This shift is indicative of increased electron density on CeO_2_ due to electron transfer from CuInS_2_ under the IEF’s influence.^68^ These changes in binding energy affirm the separation and migration of charge carriers by the S-scheme mechanism.

The band edge position and lower work function of CuInS_2_ compared to CeO_2_ implies that when in close contact, there is a spontaneous flow of electron from CuInS_2_ to CeO_2_ leading to an accumulation of positive charge on CuInS_2_ and negative charge on CeO_2_.^41^ Simultaneously, there is the creation of an internal electric field (IEF) directed from CuInS_2_ to CeO_2_. Also, the interfacial contact between the two semiconductors leads to the equilibration of the Fermi energy, which leads to band bending. The synergistic interaction of the generated IEF, band bending, and Coulombic attraction between photogenerated holes and electrons significantly enhances the migration of photogenerated electrons from the conduction band (CB) of CeO_2_ to the holes in CuInS_2_. This optimized electron transfer facilitates the availability of electrons in the conduction band of CuInS_2_ and holes in the valence band (VB) of CeO_2_, thereby promoting the degradation process. The mechanism scheme in Figure 8b was proposed on the basis of the radical scavenging experiment and the proposed S-scheme charge carrier transfer mechanism.

## Conclusion

In this study, CuInS_2_/CeO_2_ heterojunctions were synthesized by a facile hydrothermal method. The heterojunction formation at the interface led to an enhancement in the charge carrier transport and separation, which consequently improved the photocatalytic activity of the material for CIP degradation. The photocatalyst achieved a degradation efficiency of 90%, which was a significant improvement over that of the pristine material. The efficiency of the catalyst in real water samples and the stability of the material attest to its potential practical application for the treatment of wastewater from the pharmaceutical industry. Scavenging experiments showed ^•^OH, O_2_^•–^ and h^+^ played major roles in the activity of the catalyst, which were generated by the proposed S-scheme charge transfer mechanism. The revealed enhanced catalytic activity reported for CuInS_2_/CeO_2_, shows potential for application in the industrial treatment of pharmaceutical wastewater.
